# Role of Social Support in the Relationship between Sexually Transmitted Infection and Depression among Young Women in Canada

**DOI:** 10.2188/jea.JE20090133

**Published:** 2010-07-05

**Authors:** Yanhui Gao, Don MacDonald, Kayla D. Collins, Reza Alaghehbandan, Yue Chen

**Affiliations:** 1 Department of Epidemiology and Community Medicine, Faculty of Medicine, University of Ottawa, Ottawa, Ontario, Canada; 2 Department of Epidemiology and Health Statistics, School of Public Health, Guangdong Pharmaceutical University, Guangzhou, China; 3 Research and Evaluation, Newfoundland and Labrador Centre for Health Information, St. John’s, Newfoundland, Canada

**Keywords:** depression, sexually transmitted infection, social support, young women

## Abstract

**Background:**

Individuals with a self-reported history of sexually transmitted infection (STI) are at high risk for depression. However, little is known about how social support affects the association between STI and depression among young women in Canada.

**Methods:**

Data were drawn from the Canadian Community Health Survey (CCHS), conducted in 2005. A total of 2636 women aged 15–24 years who provided information on STI history were included in the analysis. Depression was measured by a depression scale based on the Composite International Diagnostic Interview Short-Form (CIDI-SF). The 19-item Medical Outcomes Study (MOS) Social Support Survey assessed functional social support. A log-binomial model was used to estimate the prevalence ratio (PR) for self-reported STI history associated with depression and to assess the impact of social support on the association.

**Results:**

The adjusted PR for self-reported STI history associated with depression was 1.61 (95% CI, 1.03 to 2.37), before social support was included in the model. The association between STI history and depression was no longer significant when social support was included in the model (adjusted PR, 1.28; 95% CI, 0.83 to 1.84). The adjusted PRs for depression among those with low and intermediate levels of social support versus those with a high level of social support were 5.62 (95% CI, 3.50 to 9.56) and 2.19 (1.38 to 3.68), respectively.

**Conclusions:**

Social support is an important determinant of depression and reduces the impact of self-reported STI on depression among young women in Canada.

## INTRODUCTION

Sexually transmitted infections (STIs) are an escalating public health challenge in Canada, and the burden of such infections is mainly borne by young women. For example, chlamydia, the most commonly reported STI in Canada, disproportionately affects women aged 15 to 24 years, who accounted for 73% of female cases and 49% of all cases in 2004.^1^

In a study of the general Canadian population, individuals with a self-reported STI history were at high risk for depression symptoms, with levels twice those of women without such a history.^2^ The consequences of depression in such patients include high comorbidity with anxiety disorders, increased risky behaviors (including risky sexual practices), substance abuse, suicide,^3^ decreased quality of life,^4^ and other negative outcomes such as immune and endocrine dysregulation.^5^^,^^6^ Some studies suggest that depression interferes with information processing, reduces motivation to change behavior, and limits the success of interventions for STI patients.^7^^,^^8^

The link between perceived social support and reduced symptoms of depression has been well established in people with various chronic illnesses^9^^,^^10^ and in HIV-positive persons.^11^^,^^12^ However, little is known of the effect of functional social support on the association between STI and depression among young women. This study used data collected from young Canadian women to test the hypothesis that social support reduces the impact of STIs on the prevalence of depression.

## METHODS

Data were drawn from the Canadian Community Health Survey (CCHS), Cycle 3.1, conducted by Statistics Canada from January through December 2005. The methodology of the survey has been described in detail elsewhere.^13^ In brief, the CCHS Cycle 3.1 used a complex multistage stratified sampling strategy to select people living in private dwellings in the 10 provinces and 3 territories of Canada. Individuals who were living on Indian Reserves or Crown lands, those residing in institutions, full-time members of the Canada Forces, and residents of certain remote regions were excluded. A total of 132 947 individuals aged 12 years or older participated in the survey, which yields a national response rate of 78.9%.

Only individuals 15–49 years of age were asked to answer the questions “Have you ever had sexual intercourse?”. Respondents who answered affirmatively to the questions “Have you ever been diagnosed with a sexually transmitted disease?” were considered to have a self-reported STI. The short-form of major depression episodes (MDE) used in the survey was based on the work of Kessler and Mroczek^14^ and assessed the depression level for respondents in the past 12 months. The items were taken from the Composite International Diagnostic Interview (CIDI), which is a structured diagnostic instrument designed to produce diagnoses according to the definitions and the criteria of Diagnostic and Statistical Manual of Mental Disorders and the diagnostic criteria for the Research of the ICD-10. Depression scores range from 0 to 8, and a higher score indicates an increased probability of a depressive disorder. Respondents with a score of 5 or higher, which corresponds to a 90% likelihood of a positive diagnosis of MDE, were classified as having had a clinical MDE in the past 12 months.^13^ For social support, the 19-item Medical Outcomes Study (MOS) Social Support Survey^15^ assessed the functional social support, including emotional or informational support (8 items), tangible support (4 items), affectionate support (3 items), and positive social interaction (4 items). It measured how often each type of support was available to respondents if they needed it (none of the time, a little of the time, some of the time, most of the time, or all of the time; indicated by a score of 0–4). For the 4 dimensions of social support and overall social support, the social support level for each individual was classified as high (“all of the time” to all items), intermediate (average score, 3 to <4), or low (average score, <3). In CCHS Cycle 3.1, the depression and social support questionnaire modules were optional. Only respondents living in the provinces of Prince Edward Island, Nova Scotia, Quebec, Saskatchewan, Alberta, and British Columbia were asked to complete the depression module; those living in the provinces of Quebec, Alberta, and British Columbia were selected to complete the social support module. As a result, only data from women aged 15–24 years who had ever had sexual intercourse and lived in Quebec, Alberta, or British Columbia were included in this analysis.

Covariates adjusted in this analysis included smoking status, alcohol use, income, educational level, major chronic conditions, and marital status. For smoking status, individuals were categorized into current smokers (daily smokers at the time of the survey), former smokers (former daily smokers who are currently nonsmokers or occasional smokers), and never smokers. Based on the frequency of alcohol use in the past 12 months, subjects were grouped into 3 categories: none or less than 2 times per week, 2 to 3 times per week, more than 3 times per week. In this survey, the level of household income for each respondent relative to that of all other respondents was expressed in terms of the household income distribution of Canadians in deciles. This takes into account the adjusted ratio of total household income to the low income cut-off corresponding to the size of both the household and the community. The income level of subjects was classified as low (lowest 30%), intermediate (middle 40%), or high (highest 30%) based on the income distribution in this analysis. Major chronic condition (yes, no) indicated whether the respondents had at least 1 major chronic health condition diagnosed by a health professional, including asthma, high blood pressure, diabetes, heart disease, cancer, stroke, chronic bronchitis, emphysema, and chronic obstructive pulmonary disease. Marital status included the categories of married/common-law partner, widowed/separated/divorced, and single.

The prevalences of self-reported STI and depression by functional social support and other covariates were calculated among women aged 15–24 years. A log-binomial regression model was used to estimate unadjusted and adjusted prevalence ratios (PRs) for self-reported STI history associated with depression and to assess the impact of social support on this association.^16^ The maximum likelihood estimations (MLEs) of PRs were obtained, and likelihood ratio confidence intervals (CIs) were used for statistical inference. Due to correlation among the 4 dimensions of social support,^15^ we fitted the log-binomial regression model using each type of social support predictor separately. All the prevalence and variance estimates accounted for the multistage, stratified, survey design. A population weight was calculated for each participant by Statistics Canada. This value indicates the number of people that he or she represents in the Canadian population. The effect of the complex survey design on variance estimates is summarized as a design effect, and the design effect is the ratio of the estimated variance based on the survey to a comparable estimate of variance from a simple random sample of the population. Standard errors were inflated by this average design effect.^17^ All the statistical analyses were conducted using SAS 9.1.

## RESULTS

Table 1 shows the crude prevalences of self-reported STI history and depression by functional social support and other covariates among women aged 15–24 years. The crude prevalence of depression in young women with a self-reported STI history was almost double that of women without such a history (21.3% versus 11.0%). Low social support was significantly associated with increased risks of both STI and depression. Women who were widowed/separated/divorced, current smokers, or who drank alcohol more than 3 times per week were at higher risk for both STI and depression. Those who had chronic conditions were also more likely to be depressed (Table 1).

**Table 1.** Prevalence of self-reported sexually transmitted infection (STI) history and depression, by functional social support and other covariates among women aged 15–24 years (Canadian Community Health Survey, 2005)*

Variables	*n*	STI		Depression	
	
		Cases	%	Cases	%
Self-reported STI history					
Yes	201			46	21.31
No	2435			234	11.02
Depression					
Yes	280	46	11.42		
No	2356	155	5.57		
Overall social support					
Low	360	57	13.09	91	30.39
Intermediate	1538	99	5.28	151	10.63
High	656	39	5.32	31	4.74
Unknown	82	6	3.12	7	6.29
Marital status					
Married/common-law ​ partner	630	55	6.69	49	8.55
Single	1982	140	6.02	227	12.51
Widowed/separated/ ​ divorced	18	5	31.71	4	22.07
Unknown	6	1	24.85	0	0
Smoking status					
Never smoker	1588	68	4.19	128	9.27
Former smoker	425	37	6.98	44	11.35
Current smoker	623	96	12.24	108	19.51
Alcohol use					
None or occasional	2165	143	5.22	225	11.40
2–3 times per week	373	37	8.42	39	12.04
≥4 times per week	91	21	20.93	14	14.92
Unknown	7	0	0	2	31.81
Income					
Low	920	100	7.74	97	11.80
Middle	811	50	5.56	93	12.32
High	394	29	9.25	34	9.75
Unknown	511	22	3.12	56	11.91
Education					
Low	577	50	5.78	79	13.28
Middle	1087	81	7.12	121	11.10
High	961	70	5.64	80	11.58
Unknown	11	0	0	0	0
Chronic condition					
Yes	450	45	6.36	67	15.52
No	2186	156	6.23	213	10.90

The adjusted PR for self-reported STI history associated with depression was 1.61 (95% CI, 1.03 to 2.37) before social support was included in the model. The associated between STI history and depression was no longer significant when overall functional social support was included in the model (adjusted PR, 1.28; 95% CI, 0.83 to 1.84). The adjusted PRs for depression among women with low and intermediate levels of overall social support versus those with a high level of social support were 5.62 (95% CI, 3.50 to 9.56) and 2.19 (1.38 to 3.68), respectively (Table 2).

**Table 2.** Adjusted prevalence ratios (PRs) and 95% confidence intervals (95% CIs) for social support, sexually transmitted infection (STI) history, and other covariates associated with depression among women aged 15–24 (Canadian Community Health Survey, 2005)

Variables	Model 1 (not including social support)	Model 2 (including social support)
Overall social support		
High		1.00
Intermediate		2.19 (1.38, 3.68)
Low		5.62 (3.50, 9.56)
Self-reported STI		
No	1.00	1.00
Yes	1.61 (1.03, 2.37)	1.28 (0.83, 1.84)
Marital status		
Married/common-law ​ partner	1.00	1.00
Single	1.52 (1.05, 2.26)	1.37 (0.96, 2.03)
Widowed/separated/ ​ divorced	1.66 (0.12, 5.09)	1.14 (0.08, 3.40)
Smoking status		
Never smoker	1.00	1.00
Former smoker	1.31 (0.86, 1.93)	1.19 (0.79, 1.74)
Current smoker	2.01 (1.47, 2.73)	1.79 (1.32, 2.41)
Alcohol use		
None or occasional	1.00	1.00
2–3 times per week	0.96 (0.64, 1.37)	0.95 (0.65, 1.33)
≥4 times per week	0.91 (0.43, 1.62)	0.79 (0.38, 1.36)
Chronic condition		
Yes	1.00	1.00
No	0.76 (0.56, 1.06)	0.83 (0.62, 1.15)

Single young women were at significantly higher risk of depression than those who were married or had a common-law partner, before controlling for social support (PR, 1.52; 95% CI, 1.05 to 2.26); however, the association was no longer significant after controlling for social support (1.37; 0.96 to 2.03). Current smokers were more likely to have depression than never smokers. The adjusted PRs for other individual characteristics were not significant either before or after including social support in the model (Table 2).

**Figure 1.**
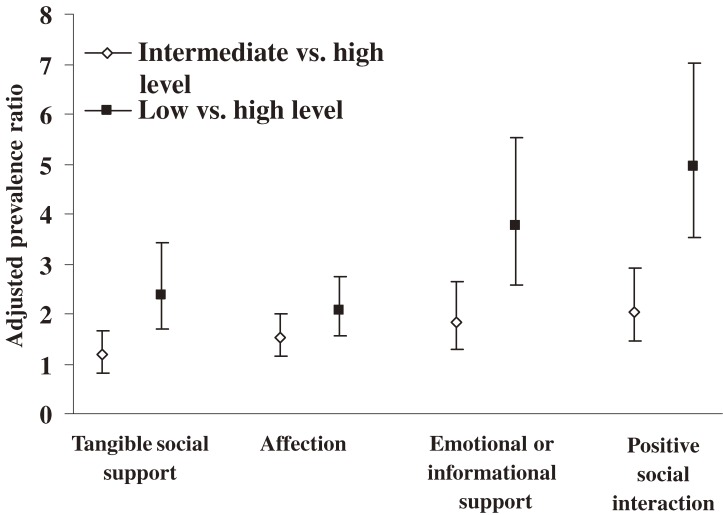
Adjusted prevalence ratios and 95% confidence intervals for depression in women aged 15–24 years with low and intermediate levels of the 4 domains of social support versus a high level of such support (Canadian Community Health Survey, 2005). Separate multiple log-binomial regression models indicated that the association between self-reported STI and depression was not significant after controlling for each type of social support.

The association between STI history and depression was also no longer significant when each of the 4 domains of social support was included in separate models (data available upon request). Positive social interaction and emotional/informational support played important roles in the association between self-reported STI history and depression (Figure 1).

## DISCUSSION

The high and increasing prevalence of STIs has a major impact on the health of Canadians. Unfortunately, women aged 15 to 24 years are overrepresented among patients with STIs and have the highest likelihood of serious and lifelong consequences of these diseases.^1^ In females, adolescence and young adulthood is an important developmental period for understanding risk behaviors and mental outcomes, because significant increases in STIs and depressive symptoms occur during this time. The association between STI and depression has been reported in a number of studies.^2^^,^^18^^–^^20^ The effect of social support on mental and behavioral outcomes among various populations has also been explored. In 1 study of 125 women and 232 men with HIV/AIDS, perceived social support was significantly associated with better mental health, as measured using indices of depression and loneliness.^11^ Adolescents with parents with HIV/AIDS who received greater social support from providers reported significantly lower levels of depression and fewer behavioral problems.^21^ In the present study, we found that the crude prevalence of depression in Canadian young women with an STI history was double that of women without such a history. Before social support was included in the analysis, the adjusted prevalence ratio for depression associated with a self-reported STI history was significantly higher; however, it was no longer significant when social support was included in the log-binomial model, suggesting that social support plays an important role in the association between STI and depression among young women in Canada.

Approximately 75% of young women aged 18 to 24 years were single in this study population. Single women were at higher risk of depression than those who were married or had a common-law relationship, but this association was no longer significant when social support was taken into consideration, indicating that increasing social support reduces the risk of depression among young women. Smoking was also significantly associated with depression; this association was not affected by social support.

The role of social support as a buffer in the context of stressors is well documented. A number of studies have shown that depression symptoms are negatively associated with functional social support among patients with chronic diseases,^9^ adolescents whose parents were infected with HIV/AIDS,^12^ and people living with HIV.^11^^,^^22^^,^^23^ For women facing the challenges of living with HIV/AIDS, social support is an important resource that is consistently correlated with enhanced psychological adaptation, as well as positive behavioral outcomes. Our findings are consistent with previous research on people living with HIV and provide further evidence that functional social support ameliorates the risk of depressive symptoms associated with STIs.

Functional social support is a multidimensional construct that encompasses tangible support, affection, positive social interaction, and emotional or informational support received from others.^15^ A study of a cohort of HIV-infected women and controls showed that women who received limited emotional and tangible support had higher levels of depressive symptoms.^24^ The present study found that perceived positive social interaction and emotional/informational social support were more important than affection and tangible social support in the association between depression and self-reported STI history among young women. These results highlight the need to encourage young females with STIs to build and strengthen reciprocal relationships by participating in volunteer and social activities with family, friends, and other persons or groups.

The mechanisms of the association between social support and symptoms of depression under stressful conditions have been explored. Stigma related to illness and the individual’s self-concept may be mediators between social support and depression when confronting a stressor. In many societies, sexual morals typically exhibit a gender imbalance. As compared with men, women infected with an STI/HIV have a stronger experience of stigma related to sexual behaviors, since women tend to be judged more harshly with respect to sexual morals.^25^ Internalized stigma was found to be a significant predictor of depression. High perceived social support from friends was associated with less perceived HIV stigma,^26^ which leads to less severe depressive symptoms.^22^ Future research is needed to assess whether perceived social support accounts for the influence of stigma on depressive symptoms among young women with STIs.

The role of social support in the association between STI and depression might also be explained by a superior self-concept, ie, self-esteem. That self-esteem is a mediator between social support and depressive symptoms has been shown in studies of patients with chronic illnesses such as rheumatoid arthritis,^27^ end-stage renal disease,^10^ cancer,^28^ and bipolar disorder,^29^ and in middle-aged and elderly people.^30^ The mediating role of self-esteem in the relationship between social support and depression has also been demonstrated among HIV-positive women.^31^ Several studies of young women have shown that a higher level of self-esteem is associated with safer sexual behavior^32^ and a lower risk of contracting STIs.^33^

A major limitation of the current study was its cross-sectional design, which precludes directional or causal interpretations. It is possible that depression can lead to a higher risk of contracting STIs and lower perceived social support. Prior research has suggested that these relationships are probably reciprocal. A prospective study design could help to identify causal relations among social support, STI, and depression. Another limitation was that the present study was based on self-reported STI history. A previous study found that social stigma and negative consequences can affect college students’ decisions to seek testing for STI.^34^ This may also have occurred among young women in the present study, which would make our analysis prone to inaccuracy and social desirability bias in STI-related estimates. In addition, the data provide no information on the type of STIs, although we know that genital chlamydia is the most commonly reported STI in Canada. The impact of different STIs on depression is likely to vary.

In conclusion, we examined the role of functional social support in the association between self-reported STI history and depression. Our findings indicate that, among young women in Canada, functional social support helps to reduce the impact of STI on depression. Further research is needed to better understand specific sources of emotional/informational support and the importance of skill in social interaction with parents, friends, peer groups, and other organizations, as well as to determine how social support can address the needs of individual with STIs. Such research could also be beneficial for professionals who hope to enhance the effectiveness of social support interventions.
